# NMDG-Functionalized UiO-66 via PEI Bridging for Efficient Boron Adsorption in Salt Lake Brine

**DOI:** 10.3390/molecules31193410

**Published:** 2026-09-24

**Authors:** Min Guo, Xunduo Wang, Leiyang Zhao, Yuping Xiao, Jiaojiao Zhang, Chengbing Ma, Qin Shuai, Shengrui Xu

**Affiliations:** 1 Qaidam Comprehensive Geological and Mineral Exploration Institute of Qinghai Province, Golmud 816099, China; guo.1027@163.com (M.G.); zhang062703@163.com (J.Z.); 18919595628@163.com (C.M.); 2 Qinghai Provincial Key Laboratory of Salt Lake Resources Exploration and Research in Qaidam Basin, Golmud 816099, China; 3 Faculty of Materials Science and Chemistry, China University of Geosciences, Wuhan 430074, China; 1202520287@cug.edu.cn (X.W.); 1202611396@cug.edu.cn (L.Z.); shuaiqin@cug.edu.cn (Q.S.)

**Keywords:** salt lake brine, N-methyl-D-glucamine, UiO-66, boron adsorption

## Abstract

Salt lake brines are a source of boron, but their complex ionic composition presents challenges for adsorption-based recovery. In this study, N-methyl-D-glucamine (NMDG) was introduced into a PEI-containing UiO-66 composite through sequential post-synthetic modification. Structural and spectroscopic characterization supported the incorporation of the organic functional layer, while the characteristic UiO-66 diffraction peaks were retained. NMDG/PEI/UiO-66 showed higher boron uptake than the tested UiO-66 and PEI/UiO-66 controls. At 298 K, the highest experimentally observed capacity was 76.11 mg/g, whereas the Langmuir-fitted limiting capacity was 97.59 mg/g. The kinetic and equilibrium data were described by the pseudo-second-order and Langmuir models, respectively. The material retained approximately 86% of its initial adsorption capacity after three adsorption–desorption cycles and showed boron uptake in brines from Qarhan and Mami Cuo salt lakes. FTIR and XPS observations were consistent with the involvement of the functional layer in boron uptake. These results demonstrate the potential of the NMDG-containing UiO-66 composite for boron recovery under the investigated conditions.

## 1. Introduction

Boron, as a strategic non-metallic resource, possesses irreplaceable application value in high-tech fields such as aerospace, the nuclear industry, specialty glass, new energy, and modern agriculture due to its unique physicochemical properties [1,2]. With the rapid development of global emerging industries, the demand for high-quality boron resources has increased dramatically. Traditional solid boron mining faces challenges including high costs, environmental pollution, and high energy consumption, whereas salt lake brines contain extremely abundant boron resources [3]. Extraction of boron from salt lake brines represents a crucial strategy for overcoming these bottlenecks, as it can not only effectively reduce production costs and environmental impact but also enhance the comprehensive utilization efficiency of resources [4,5]. However, salt lake brines are typically high-salinity, multicomponent systems in which boron commonly occurs as low-concentration boric acid or borate salts [6]. Therefore, appropriate methods are required to efficiently and selectively extract boron from brine.

Current technologies for boron extraction from salt lake brines mainly include electrocoagulation [7], chemical precipitation [8], membrane separation [9], ion exchange [10], and adsorption [11]. Among these, the adsorption approach is considered one of the most promising approaches for industrial application due to its operational simplicity, environmental friendliness, low energy consumption, and good reusability [12]. The adsorption process essentially relies on the enrichment of boron species onto the adsorbent surface through physical or chemical interactions; therefore, the design of the adsorbent is the key to achieving efficient boron separation. However, conventional boron adsorbents, such as layered double hydroxides, inorganic oxides, and commercial boron-specific resins, generally suffer from drawbacks including low adsorption capacity, insufficient selectivity, and poor structural stability in high-salinity salt lake brines. These limitations make it difficult to meet the requirements for efficient boron separation from complex brine systems. Metal–organic frameworks (MOFs) have demonstrated tremendous potential in adsorptive separation and other applications owing to their ultra-high specific surface areas, tunable pore structures, and abundant functionalization sites [13,14,15]. In particular, UiO-66, which features zirconium (Zr) as the metal center, exhibits exceptional chemical stability, thermal stability, and water resistance due to the high coordination strength of its Zr–O bonds [16]. However, it is difficult to observe sufficient adsorption capacity towards boron by pristine UiO-66. To enhance performance, its facile functionalization should be leveraged by introducing appropriate functional groups. Modifying the linker or introducing functional groups into the UiO-66 framework has been demonstrated as an effective strategy to tune its adsorption selectivity toward target species [17]. Moreover, the robust framework of UiO-66 enables it to serve as a suitable carrier for boron adsorption in high-salinity brines [18].

Previous studies demonstrate that hydroxyl-rich surface functionalization is a key strategy for boron removal due to the fact that boric acid readily esterifies with cis-diol groups [19]. N-Methyl-D-glucamine (NMDG) provides abundant cis-diols that enable stable cyclic borate ester formation. Its tertiary amines synergistically enhance boron uptake, with protonation in a neutral medium electrostatically attracting B(OH)_4_^−^ and deprotonation in an alkaline medium accelerating esterification [19,20,21]. Hence, modifying UiO-66 with NMDG using an appropriate strategy is an effective approach to achieve specific boron adsorption in brine. Studies have shown that ECH and the chlorinated intermediate CI-NMDG formed by the epoxidation-opening reaction of NMDG can further undergo nucleophilic substitution reactions with amine compounds. Based on this, UiO-66 was first silanized with KH550 and then grafted with hyperbranched polyethyleneimine (PEI) to maximize surface amine sites. Subsequent nucleophilic substitution with Cl-NMDG yielded a composite adsorbent with highly specific boron recognition ability [22,23,24].

In this study, a novel porous composite adsorbent (NMDG/PEI/UiO-66) was designed and prepared via a post-synthetic modification strategy for the efficient adsorption of boron from aqueous solutions. First, PEI, with its abundant amino groups, was employed as a bridge to connect to the UiO-66 framework, followed by the successful grafting of hydroxyl-rich NMDG. The surface morphology and chemical structure of the synthesized adsorbents were characterized using scanning electron microscopy (SEM), Fourier-transform infrared spectroscopy (FTIR), powder X-ray diffraction (XRD), thermogravimetric analysis (TGA), and X-ray photoelectron spectroscopy (XPS). From a practical perspective, the evaluation of boron adsorbents for salt lake brines should not rely solely on equilibrium capacity. pH compatibility, regeneration stability, solid–liquid separation, selectivity, and performance in realistic brine matrices are equally important [25]. Therefore, the adsorption performance of NMDG/PEI/UiO-66 was evaluated not only through thermodynamic and kinetic experiments but also through interference tests, regeneration experiments, and real-brine tests. The study provides a novel functionalized composite material for the efficient adsorption of boron from brines.

## 2. Materials and Methods

### 2.1. Reagents and Materials

N,N-Dimethylformamide (DMF, 99.5%) and boric acid (H_3_BO_3_, 99.5%) were purchased from Shanghai Maclin Biochemical Technology Co., Ltd. (Shanghai, China). Chloro-zirconium (ZrCl_4_, 98%), terephthalic acid (BDC, 98%), sodium hydroxide (98%), polyethyleneimine (PEI, 99%, M.W. 600), and N-methyl-D-glucamine (NMDG, 99%) were purchased from Shanghai Aladdin Biochemical Technology Co., Ltd. (Shanghai, China) 3-Aminopropyltriethoxysilane (KH550/APTES, 97%) was purchased from Shanghai Saen Chemical Technology Co., Ltd. (Shanghai, China). All reagents were used without further purification.

### 2.2. Overview of Synthesis

The overall preparation of NMDG/PEI/UiO-66 is illustrated in Figure 1. The synthesis involved three main steps: (i) solvothermal synthesis of the UiO-66 framework; (ii) silanization with KH550 followed by PEI grafting onto the pore channels and surface of UiO-66; and (iii) anchoring of NMDG onto the PEI-modified UiO-66 through a nucleophilic substitution reaction.

### 2.3. Synthesis of UiO-66

UiO-66 was synthesized using the hydrothermal method reported in the literature [26], with some minor modifications. ZrCl_4_ (190.30 mg, 0.80 mmol) and 1,4-benzoic acid (BDC, 133 mg, 0.80 mmol) were dissolved in 60 mL N,N-dimethylformamide (DMF), and 7.25 g acetic acid (120 mmol) was added as a modifier. The mixture was stirred continuously for 2 h and then ultrasonically dispersed for 30 min. It was transferred to a 100 mL stainless steel high-pressure reactor with a polytetrafluoroethylene inner lining and reacted at 120 °C for 24 h. The solid product was collected by centrifugation and then washed three times with DMF and anhydrous methanol, respectively, to remove residual reactants and replace the solvents. The obtained sample was vacuum-dried at 60 °C for 12 h, resulting in a white powder of UiO-66.

### 2.4. Synthesis of PEI/UiO-66

Briefly, 1 g of fully dried UiO-66 powder was ground and set aside. Separately, KH-550 (0.05 g, 0.23 mmol) was added to 50 mL of ethanol and stirred magnetically in a water bath at 55 °C for 5 h to achieve complete hydrolysis. The hydrolyzed KH-550 solution was then mixed with the ground UiO-66 powder and stirred for another 4 h to allow sufficient contact and silanization between KH-550 and UiO-66. The resulting mixture was dried in a vacuum oven, ground with anhydrous ethanol, washed, centrifuged, and dried again to yield the amino-functionalized intermediate KH-550/UiO-66. Subsequently, PEI (0.2 g, 0.33 mmol) was dissolved in 30 mL of methanol, and the KH-550/UiO-66 powder was added. The reaction mixture was stirred for 12 h [27]. After the reaction, the product was collected by vacuum drying, affording the polyethyleneimine-modified composite, denoted as PEI/UiO-66.

### 2.5. Synthesis of NMDG/PEI/UiO-66

The process of polyol grafting was carried out based on the methods reported in the literature and was further improved [22]. Briefly, 9.55 g (48.9 mmol) of N-methyl-D-glucamine (NMDG) and 4.15 mL (53 mmol) of epichlorohydrin (ECH) were dispersed in 30 mL of DMF. The mixture was stirred at 318 K for 24 h to obtain the polyol reaction solution. Then, 20 mg of PEI/UiO-66 was dispersed in 20 mL of the above polyol reaction solution and ultrasonicated for 30 min. During this process, 2 mL of triethylamine was added dropwise. The reaction system was heated to 353.15 K and reacted for 24 h. After the reaction, the mixture was washed multiple times with deionized water and anhydrous ethanol and then dried in a vacuum at 60 °C overnight to obtain the final product, NMDG/PEI/UiO-66.

### 2.6. Adsorption Experiments

All the boric acid adsorption experiments were conducted by batch adsorption. Before conducting the adsorption experiment, the prepared adsorbent needs to undergo vacuum drying and activation to remove the residual solvents and reactant molecules from the material framework. The samples were placed in a vacuum drying oven and activated under vacuum at 60 °C for 12 h. The concentration of boron during the adsorption process was determined by ICP-OES, and the calculation formula for the adsorption amount is shown in (1).

The calculation process of adsorption capacity Q is shown in Equation (1):

(1) $$q_{t}\;=\;\frac{(C_{0}-C_{t})V}{M}$$

where q represents the adsorption capacity (mg/g), C_0_ is the concentration of boric acid solution before adsorption (mg/L), C_t_ is the concentration of boric acid solution after adsorption time t (mg/L), M (g) represents the mass of the adsorbent, and V (L) represents the volume of the adsorption liquid.

## 3. Results and Discussion

### 3.1. Preparation and Characterization of Adsorbent

The morphology of pristine UiO-66 and NMDG/PEI/UiO-66 was characterized using SEM. As shown in Figure 2a, pristine UiO-66 exhibited a regular octahedral crystal morphology with sharp edges and uniform particle size, consistent with typical UiO-66 features [28]. After modification by NMDG (Figure 2b), the composite still retained an intact three-dimensional polyhedral framework structure, with no obvious structural collapse or morphological change observed. This indicates that the mild grafting reaction conditions effectively maintained the structural integrity of UiO-66, providing structural assurance for the pore characteristics and mass-transfer properties in the subsequent adsorption process.

To evaluate the impact of the functionalization process on the crystal structure of UiO-66, XRD characterization was performed on both pristine UiO-66 and NMDG/PEI/UiO-66 (Figure 2c). The XRD patterns illustrate that the characteristic diffraction peaks of NMDG/PEI/UiO-66 after polyol functionalization at 7.40°, 8.50°, and 25.80° are in good agreement with the simulated standard pattern of UiO-66 reported in the literature and the pattern of the as-synthesized pristine UiO-66 [29,30,31,32]. These results demonstrate that, despite the multi-step surface chemical modification, the topological framework structure of UiO-66 was completely preserved, and the subsequent silanization, PEI coating, and NMDG grafting reactions did not compromise its crystalline order. Furthermore, the modification process did not disrupt the crystal structure of UiO-66, and the as-prepared adsorbent retained respectable crystallinity.

FTIR was used to characterize and comparatively analyze the samples before and after modification (Figure 2d), further confirming the successful loading of NMDG onto the UiO-66 framework. For pristine UiO-66, distinct absorption peaks were observed at 1580 cm^−1^ and 1394 cm^−1^, attributed to the asymmetric and symmetric stretching vibrations of the carboxyl groups in the terephthalic acid ligand, respectively [33]. The absorption peaks below 750 cm^−1^ are attributed to characteristic vibrations of Zr-O bonds and the phenyl ring of the ligand, confirming the presence of the typical UiO-66 framework [26]. After NMDG modification, the NMDG/PEI/UiO-66 composite retains all these framework peaks, demonstrating that the MOF crystal structure remains intact. Additionally, a markedly enhanced and broad absorption band appears in the 3000–3600 cm^−1^ region, which is assigned to O-H stretching vibrations of the abundant hydroxyl groups that are introduced by NMDG [34]. Strong intra- and intermolecular hydrogen bonding accounts for the significant broadening [35]. Collectively, the preserved UiO-66 fingerprint peaks and the intensified hydroxyl band in the FTIR spectra confirm the successful modification of UiO-66 with NMDG.

The N_2_ adsorption–desorption isotherms and pore size distribution of NMDG/PEI/UiO-66 (Figure 2e,f) give a BET surface area of 775.5 m^2^/g and an average pore diameter of 2.44 nm, consistent with a mesoporous structure. The relatively large surface area and mesoporous characteristics may provide accessible sites and may facilitate mass transfer for boron adsorption. Although functionalization often reduces the specific surface area of UiO-66, the adsorption capacity toward target species can be enhanced when specific functional groups are introduced. Similar behavior has been reported for UiO-66-based adsorbents, in which amino groups altered the adsorption selectivity and capacity toward different guest molecules [36].

The thermal stability of NMDG/PEI/UiO-66 was evaluated by thermogravimetric analysis (TGA) under a nitrogen atmosphere with a heating rate of 10 °C/min (Figure S1). The TG and DTG curves exhibited multi-stage weight loss characteristics. An initial weight loss of approximately 15–16% occurred below 120 °C, accompanied by a DTG peak at 61.4 °C, which is attributed to the release of adsorbed water and residual volatiles in the porous composite. Upon further heating, the mass gradually decreased, followed by entry into a broad degradation region with a DTG peak at 434.3 °C. This high-temperature process may stem from the overlapping decomposition of grafted PEI/NMDG groups and the organic linkers of the UiO-66 framework. Within the temperature range of 33.2–797.9 °C, the cumulative weight loss reached 68.37%, with a residual mass of 31.36% at 797.9 °C. The residue likely contains inorganic zirconium species as well as carbonaceous products formed under nitrogen. The TGA results indicate that NMDG/PEI/UiO-66 exhibits adequate thermal stability over the temperature range relevant to adsorption applications.

### 3.2. Adsorption Performance of NMDG/PEI/UiO-66 Towards Boron

The adsorption capacities of pristine UiO-66, PEI/UiO-66, and NMDG/PEI/UiO-66 were compared under identical conditions (Figure 3a). Pristine UiO-66 showed a capacity of 21.82 mg/g. After PEI introduction, the capacity remained almost unchanged (23.51 mg/g), indicating that PEI alone does not significantly enhance boron adsorption. After further NMDG grafting, the capacity increased markedly to 41.66 mg/g. This stepwise comparison confirms that the NMDG-derived cis-diol groups are the main contributors to the enhanced boron uptake, while PEI mainly serves as a bridging and amine-functionalized support for NMDG grafting [19].

Solution pH affects both the speciation of boron in solution and the protonation state of the functional groups on the adsorbent surface. The effect of the initial pH (3–11) on the adsorption performance of NMDG/PEI/UiO-66 is shown in Figure 3b. The adsorption capacity first increased and then slightly decreased with increasing pH, reaching a maximum at pH 9.

This behavior can be understood from the combined changes in the functional layer and boron speciation. As the pH increases from acidic to mildly alkaline conditions, the NMDG amine groups gradually deprotonate, which increases the electron density and nucleophilicity of the adjacent cis-diol groups and thereby favors esterification with boron species. At the same time, B(OH)_4_^−^ becomes increasingly available and is more reactive toward cis-diol groups, readily forming stable five- or six-membered cyclic borate esters. The maximum adsorption at pH 9 therefore arises from a favorable combination of a more nucleophilic polyol environment and an increased contribution from B(OH)_4_^−^. When the pH is further increased to 11, deprotonation of the cis-diol groups and a more negative surface charge hinder the approach of borate ions, leading to a decrease in adsorption [23]. These effects may be further modulated by ionic strength under high-salinity conditions, which is relevant to real brine applications.

### 3.3. Adsorption Kinetics

As shown in Figure 4a, NMDG/PEI/UiO-66 exhibited a relatively fast adsorption rate during the first hour. This fast adsorption was attributed to the abundant accessible cis-diol groups and Zr-O unsaturated sites on the surface and within the pores of the material. Adsorption equilibrium was nearly reached after 2 h. To reveal the adsorption mechanism and rate-determining step, the experimental data were fitted using pseudo-first-order and pseudo-second-order kinetic models (Figure 4b,c and Table S1). The kinetic results indicate that the correlation coefficient (R^2^) of the pseudo-second-order model reaches 0.997, which is markedly higher than that of the pseudo-first-order model (R^2^ = 0.600). This suggests that chemisorption plays a dominant role in the adsorption of boron onto NMDG/PEI/UiO-66.

### 3.4. Adsorption Isotherms and Thermodynamics

To investigate the effects of temperature and initial concentration on boron adsorption by NMDG/PEI/UiO-66, batch experiments were carried out at 298, 308, and 318 K with initial boron concentrations ranging from 50 to 2000 mg/L. As shown in Figure 5a, the adsorption capacity increased with rising concentration and reached a maximum at 1500 mg/L. At 298 K, the maximum experimentally observed capacity was 76.11 mg/g at 1500 mg/L. This value is distinct from the Langmuir-fitted theoretical maximum capacity of 97.59 mg/g at 298 K (Table S2). The capacity decreased markedly as the temperature increased, indicating that the adsorption process was exothermic. To investigate the adsorption behavior at different temperatures, two typical isotherm models, Langmuir and Freundlich, were applied. The fitting results are shown in Figure 5b,c, and the corresponding parameters are listed in Table S2. The Langmuir model provided a better fit than the Freundlich model, which is consistent with a Langmuir-type monolayer adsorption description. This observation is in line with the intended material design, in which NMDG-derived cis-diol groups are grafted onto the PEI branches and may complex with boric acid/borate species.

Thermodynamic analysis provided further insight into the adsorption energetics. Using the Van’t Hoff equation with a dimensionless equilibrium constant referenced to a defined standard state, the standard enthalpy change (ΔH^0^) and entropy change (ΔS^0^) were estimated, and the standard Gibbs free energy change (ΔG^0^) was obtained from the linear relationship between ln K^0^ and 1/T (Figure 5d and Table S3). The negative ΔH^0^ value is consistent with the exothermic nature of the adsorption, in line with the decreased capacity observed at higher temperatures. The negative ΔS^0^ value (−53.56 J mol^−1^ K^−1^) indicates a net decrease in entropy at the solid–liquid interface upon boron binding. In a grafted PEI/NMDG layer, such an entropy change may also include contributions from hydration-shell rearrangement and conformational ordering of the grafted chains [37].

Table 1 summarizes the boron adsorption performance of selected adsorbents. Under the conditions examined in this study, NMDG/PEI/UiO-66 achieved a maximum experimentally observed capacity of 76.11 mg/g at an initial boron concentration of 1.5 g/L, pH 9, and 25 °C. Because the literature values were obtained under different concentrations, pH values, and temperatures, direct comparisons of adsorption capacities should be made with caution. To further assess the potential of this composite for boron recovery from salt-lake brines, its tolerance to coexisting ions, regeneration behavior, and adsorption performance in real brines were subsequently investigated.

### 3.5. Effect of Interference Substances and Reusability Performance

The main coexisting substances in the actual brine matrix include K^+^, Mg^2+^, Ca^2+^, SO_4_^2−^, CO_3_^2−^, NO_3_^−^. To evaluate the adsorption performance of the prepared adsorbent in real brine, the effects of these interfering substances were investigated. As shown in Figure 6a, the presence of Mg^2+^ and K^+^ had no significant effect on the adsorption capacity under the tested equal-mass-concentration conditions. Ca^2+^ slightly promoted adsorption, with the capacity reaching approximately 49 mg/g, which may be attributed to compression of the electrical double layer by appropriate ionic strength, weakening the electrostatic repulsion between B(OH)_4_^−^ ions. In contrast, NO_3_^−^ and SO_4_^2−^ caused an approximately 30% decrease in adsorption capacity, which may be related to competition between these anions and B(OH)_4_^−^-protonated amino sites and the generation of steric hindrance. CO_3_^2−^ was associated with the largest decrease, causing the adsorption capacity to drop sharply to approximately 21 mg/g. This may be because the hydrolysis of CO_3_^2−^ increases the solution pH, leading to competition between OH^−^ and B(OH)_4_^−^ for adsorption sites and promoting deprotonation of amino groups on the PEI chains, which may weaken the electrostatic auxiliary attraction.

The desorption and regeneration performance of NMDG/PEI/UiO-66 was also evaluated. 0.5 M NaOH was used as the desorption reagent because alkaline conditions promote the hydrolysis of borate esters and convert boron to B(OH_4_)^−^, thereby facilitating desorption. The desorption conditions were a solid-to-liquid ratio of 1 g/L and a desorption time of 2 h. After three adsorption–desorption cycles, the material retained approximately 85.93% of its initial adsorption capacity. The capacity decreased by only 3.3% in the second cycle, indicating effective regeneration in the first cycle. The capacity loss increased to 14.07% in the third cycle, possibly due to the accumulation of residual boron on some active sites or the detachment of a small amount of grafted NMDG during repeated acid–base treatments. After three cycles, the material still retained over 85% of its initial capacity, indicating that NMDG/PEI/UiO-66 can be regenerated and reused, which is favorable for practical boron extraction from salt-lake brines.

### 3.6. Adsorption Mechanism

To further investigate the adsorption mechanism of boron on NMDG/PEI/UiO-66, the material was characterized by FTIR before and after adsorption. As shown in Figure 7a, the composite retained the asymmetric and symmetric stretching vibration peaks of carboxyl groups (-COO^−^) near 1580.40 and 1396.76 cm^−1^, as well as the Zr-O cluster vibration peaks in the 500–700 cm^−1^ range [28,39], indicating that the main UiO-66 framework was largely retained after boron adsorption. Compared with the spectrum before adsorption, the broad hydroxyl (-OH) stretching band at approximately 3400 cm^−1^ weakened after boron adsorption, which is consistent with the participation of surface hydroxyl groups in the adsorption process. In the spectrum after adsorption, a distinct band appeared in the 1050–1100 cm^−1^ region, consistent with the formation of B-O-C bonds [40]. These observations suggest that boric acid/borate species interact with the cis-diol groups of grafted NMDG to form borate esters. This is consistent with the kinetic results, which point to a chemisorption-dominated process.

XPS analysis was performed on NMDG/PEI/UiO-66 before and after adsorption. The survey spectra (Figure 7b) showed C, N, O, and Zr in both states. Before adsorption, the C 1s, O 1s, Zr 3d, and N 1s peaks were clearly observed, consistent with the expected UiO-66 framework and successful NMDG grafting [41]. After adsorption, a new B 1s peak appeared at approximately 191.52 eV [42], indicating boron enrichment on the surface. The Zr 3d peaks remained visible in both states, indicating that Zr remained in the solid phase and that no obvious framework collapse occurred.

High-resolution O 1s spectra (Figure 7c) provided further insight into the bonding changes. Before adsorption, the O 1s envelope was deconvoluted into Zr-O, C-O, and surface -OH components. After boron adsorption, a B-O component emerged, accompanied by slight shifts in the relative proportions and binding energies of the -OH and C-O peaks, suggesting that surface hydroxyl groups are involved in boron binding. The multiple hydroxyl oxygens of NMDG can act as nucleophiles toward the electron-deficient boron center to form borate esters. In the N 1s spectrum (Figure 7d), the free amine and protonated amine peaks shifted to 399.17 and 401.85 eV after adsorption, which may reflect changes in the electron density around nitrogen and possible interactions between the amine groups and boron species. Meanwhile, the Zr 3d_5/2_ and Zr 3d_3/2_ binding energies shifted from 181.08 and 183.88 eV to 181.90 and 184.45 eV, respectively [43]. This shift suggests a change in the electron density around Zr, and boric acid may also interact with the Zr-O clusters of UiO-66 [44,45].

Evidence from FTIR and XPS points to boron enrichment on NMDG/PEI/UiO-66, with surface hydroxyl groups, amine groups, and Zr-O clusters potentially all involved in the adsorption process. Borate ester formation between boric acid/borate species and the cis-diol groups of NMDG is supported by the FTIR band at 1050–1100 cm^−1^ and the changes in the O 1s components. Further interactions involving the amine groups and Zr-O clusters are suggested by the shifts in the N 1s and Zr 3d binding energies. Zr remains in the solid phase, as indicated by the Zr 3d signal. A synergistic multi-site mechanism, rather than a single uniquely defined bonding pathway, is therefore favored by the overall data.

### 3.7. Adsorption Performance of NMDG/PEI/UiO-66 for Boron in Real Brine

Two real brines, collected from Qarhan Salt Lake in Qinghai and Mami Cuo Salt Lake in Tibet, were used to evaluate the performance of NMDG/PEI/UiO-66 under realistic conditions. The physicochemical parameters of the two brines are summarized in Table S4. Compared with pure boric acid solutions, the boron adsorption capacity decreased in both brines (Figure 8), likely due to their high salt contents and complex ionic compositions, which may occupy adsorption sites and compete with boron species. Despite this decrease, NMDG/PEI/UiO-66 still showed boron uptake in both brines, indicating boron uptake in these complex matrices.

The difference between the two brines can be attributed to variations in boron concentration, pH, and coexisting ions. Mami Cuo brine has a higher boron concentration (360.45 mg/L) and a pH of 8.95, which is close to the apparent optimum for boron adsorption by this material. In contrast, Qarhan brine has much higher TDS (420.09 g/L), Cl^−^ concentration (306.54 g/L), and Mg^2+^ concentration (101.46 g/L), which may contribute to stronger competitive and salting-out effects. As a result, the boron adsorption capacity reached approximately 41 mg/g in Mami Cuo brine, whereas it was approximately 23 mg/g in Qarhan brine. These results suggest that NMDG/PEI/UiO-66 has potential for boron recovery from real brines while also indicating that matrix effects should be considered in practical applications.

## 4. Conclusions

In this study, an NMDG/PEI/UiO-66 composite porous adsorbent was prepared through a polyol functionalization strategy, which enhanced the boron complexation ability of the UiO-66 support. The material retained the structural stability of the UiO-66 framework and exhibited fast adsorption kinetics, improved boron capacity, and good tolerance to coexisting ions under the tested conditions. The adsorption data were well described by the pseudo-second-order kinetic and Langmuir isotherm models, consistent with chemisorption-dominated uptake involving the cis-diol groups of NMDG. Regeneration tests and real brine experiments indicated that the adsorbent can be reused and remains effective in complex, high-salinity matrices. These results suggest that NMDG/PEI/UiO-66 is a promising candidate for boron recovery from salt-lake brines and provide useful insights for the design of polyol-functionalized adsorbents.

## Figures and Tables

**Figure 1:**
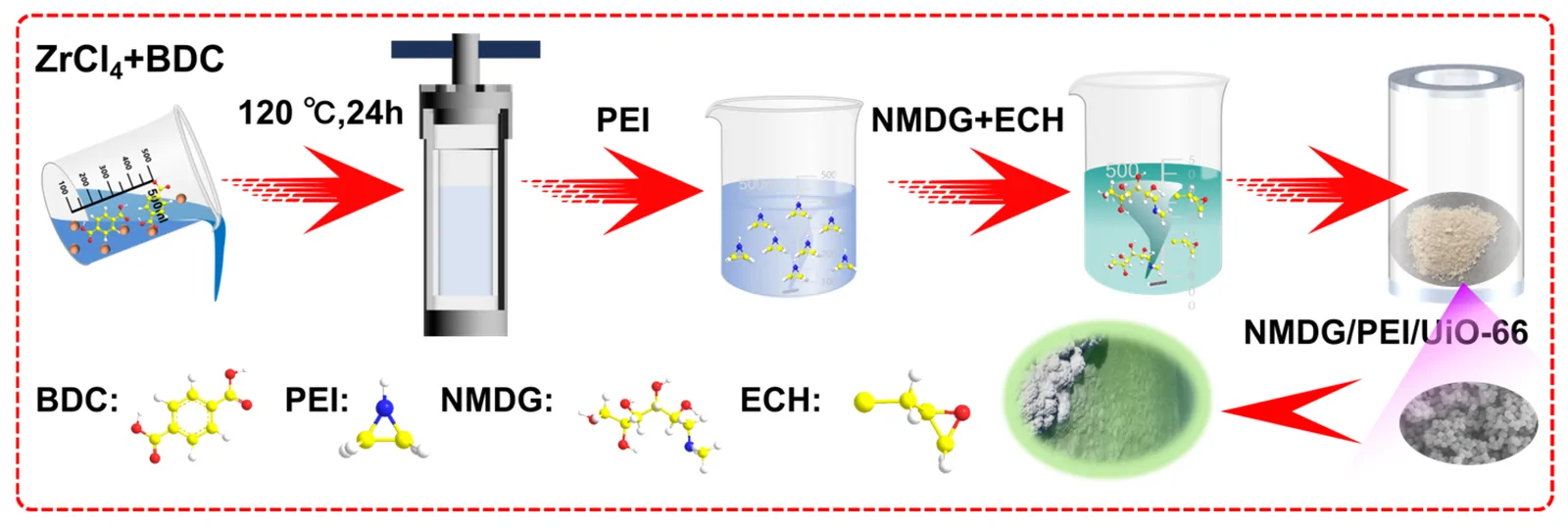
Schematic illustration of the synthesis process of NMDG/PEI/UiO-66.

**Figure 2:**
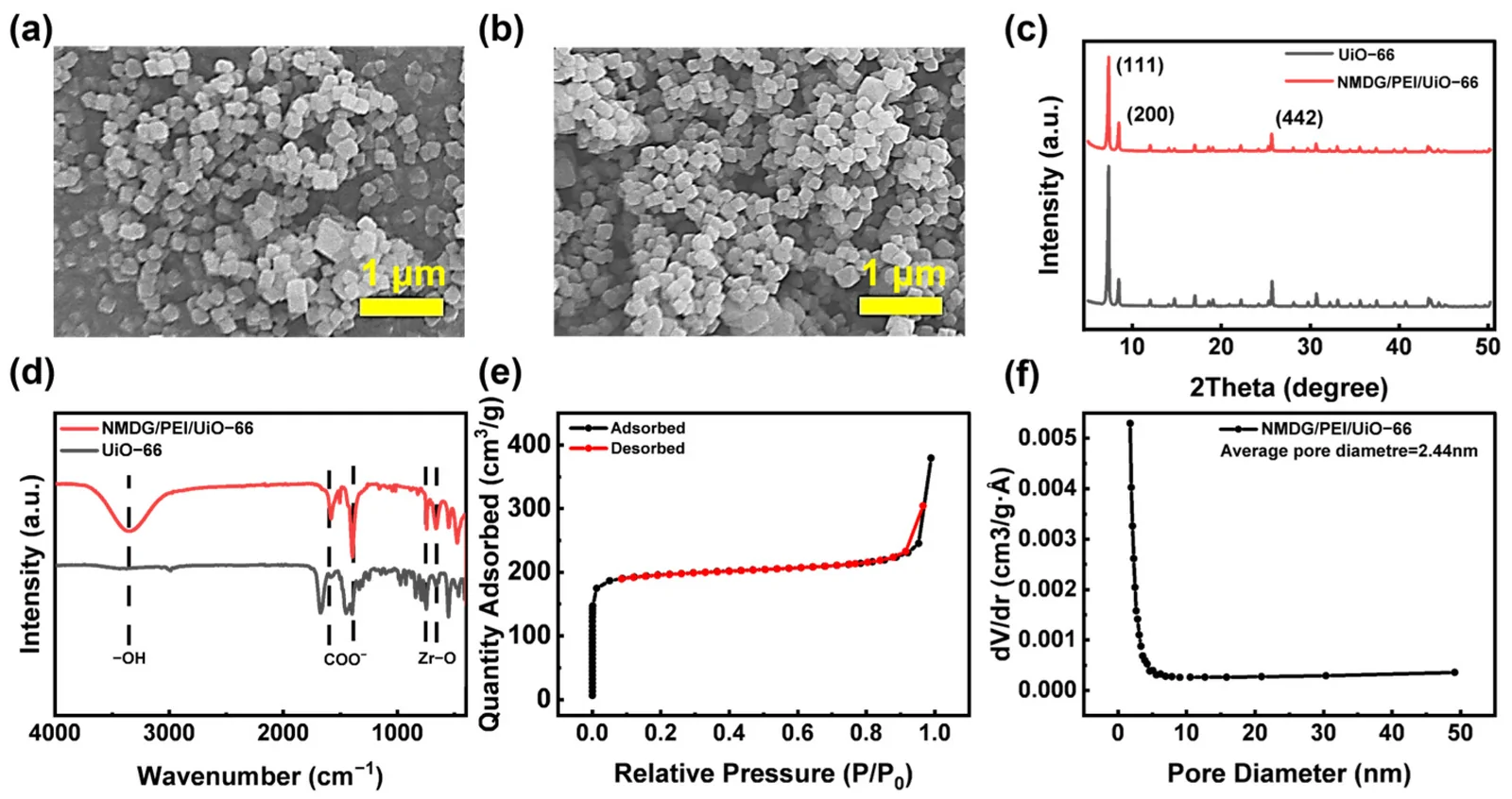
(**a**,**b**) SEM images of UiO-66 and NMDG/PEI/UiO-66. (**c**) XRD patterns of UiO-66 and NMDG/PEI/UiO-66. (**d**) FT-IR spectra of UiO-66 and NMDG/PEI/UiO-66. (**e**) N_2_ adsorption–desorption isotherm of NMDG/PEI/UiO-66. (**f**) Pore size distribution of NMDG/PEI/UiO-66.

**Figure 3:**
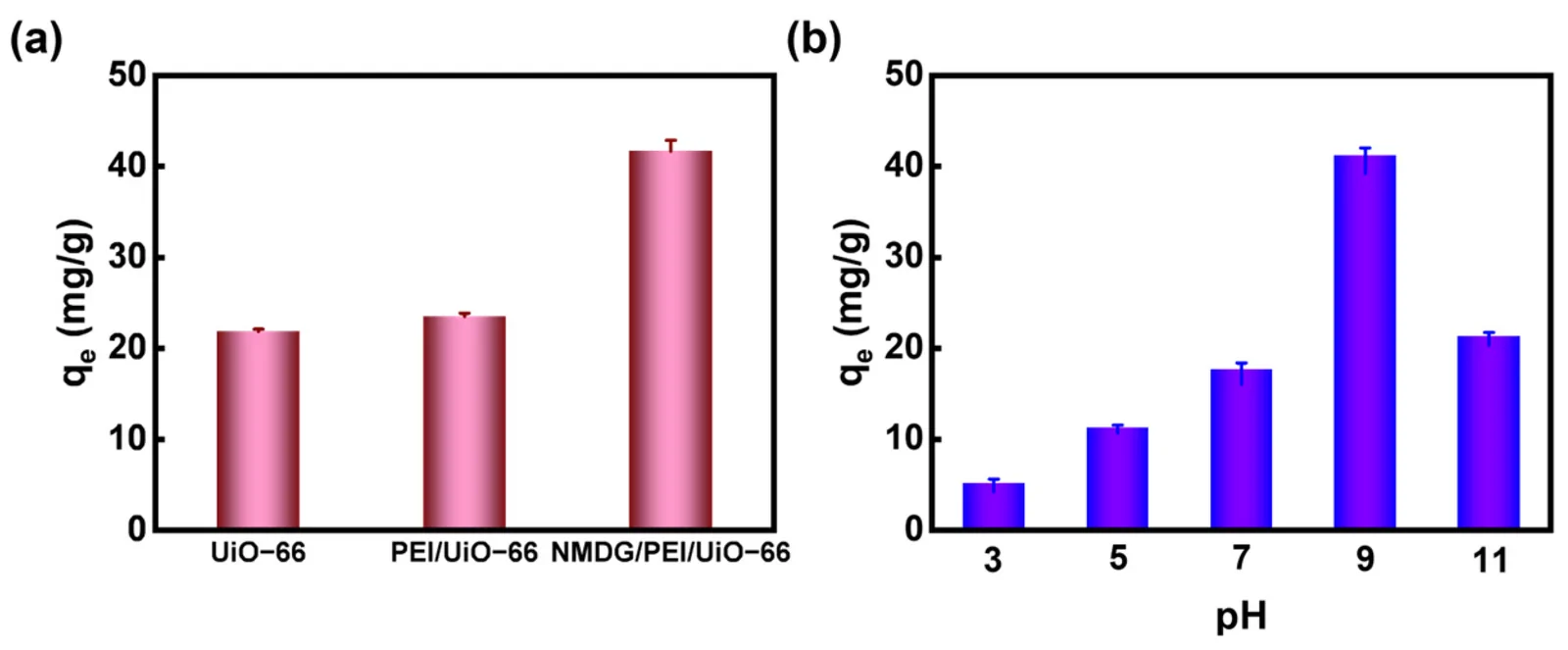
(**a**) Adsorption capacity of pristine UiO-66, PEI/UiO-66, and NMDG/PEI/UiO-66 for boron at 200 mg/L. (**b**) Effect of pH on adsorption of NMDG/PEI/UiO-66 at different pH values.

**Figure 4:**
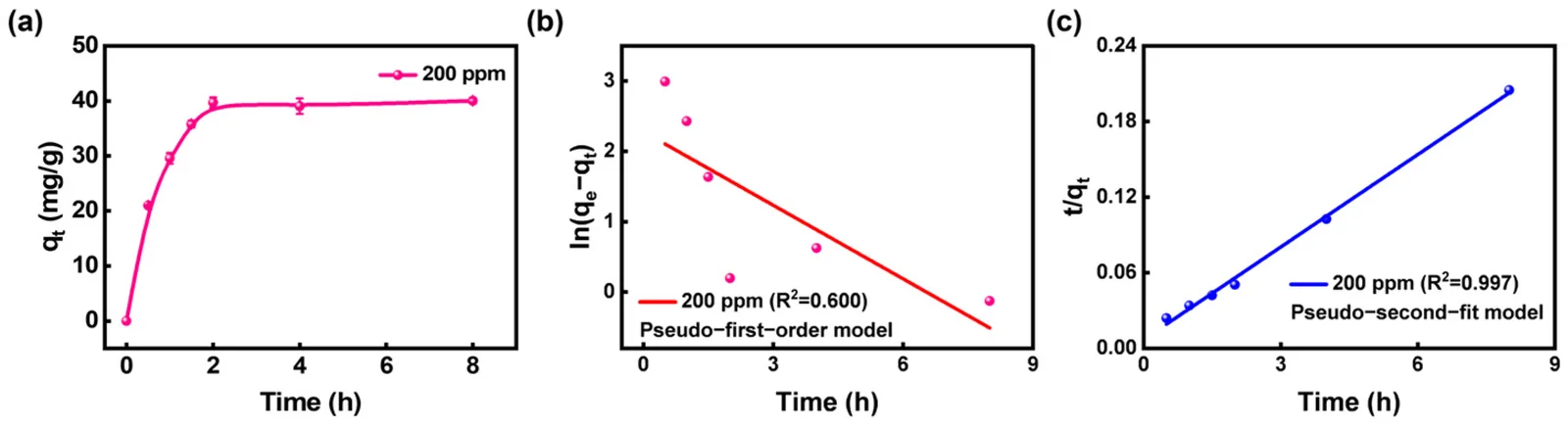
(**a**) Adsorption of NMDG/PEI/UiO-66 at different time intervals. (**b**) Pseudo-first-order kinetic simulation results for NMDG/PEI/UiO-66. (**c**) Pseudo-second-order kinetic fitting results.

**Figure 5:**
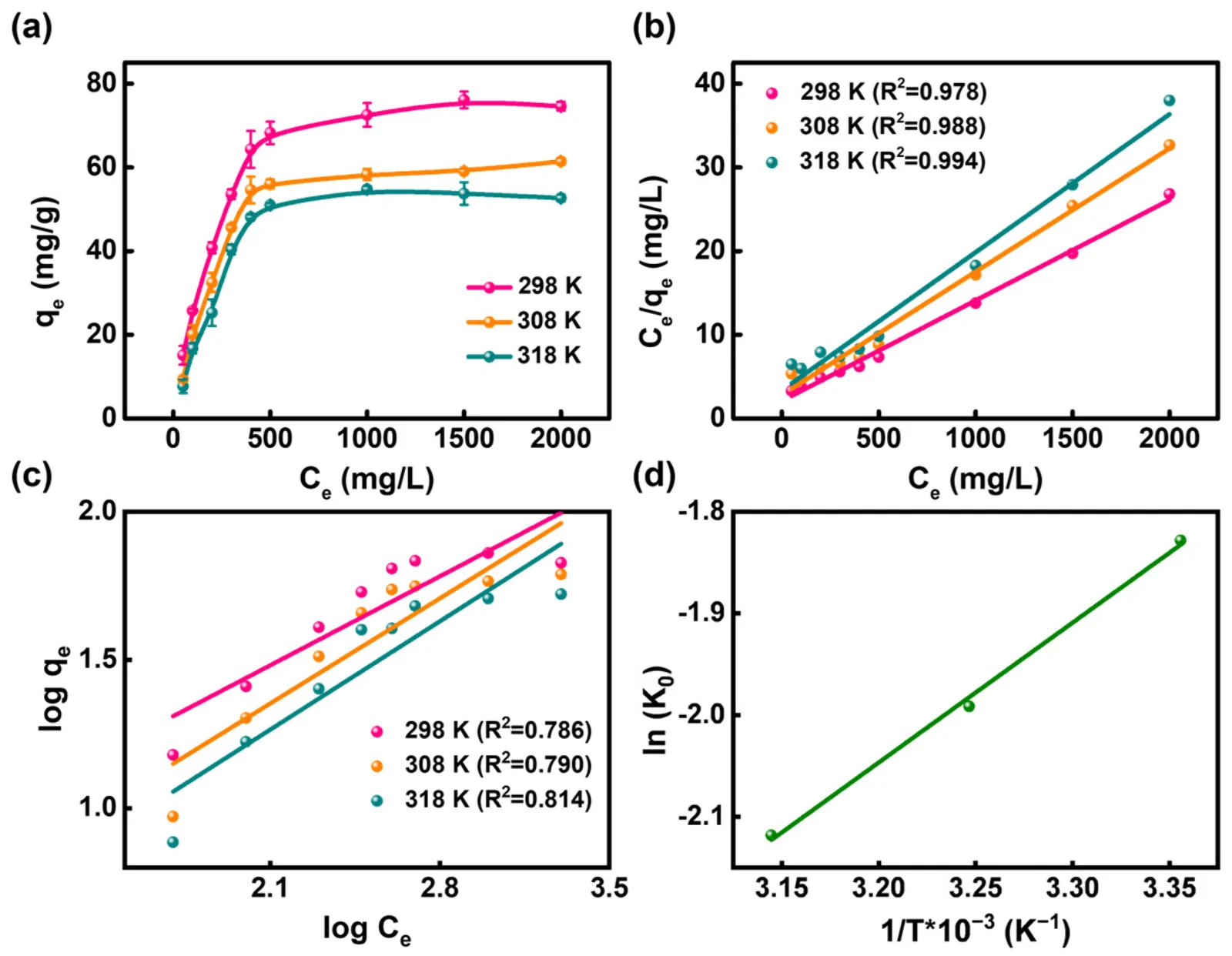
(**a**) Adsorption isotherms of NMDG/PEI/UiO-66 at different temperatures. (**b**) Langmuir-fitted adsorption isotherm for NMDG/PEI/UiO-66. (**c**) Freundlich-fitted adsorption isotherm for NMDG/PEI/UiO-66. (**d**) Van’t Hoff plot.

**Figure 6:**
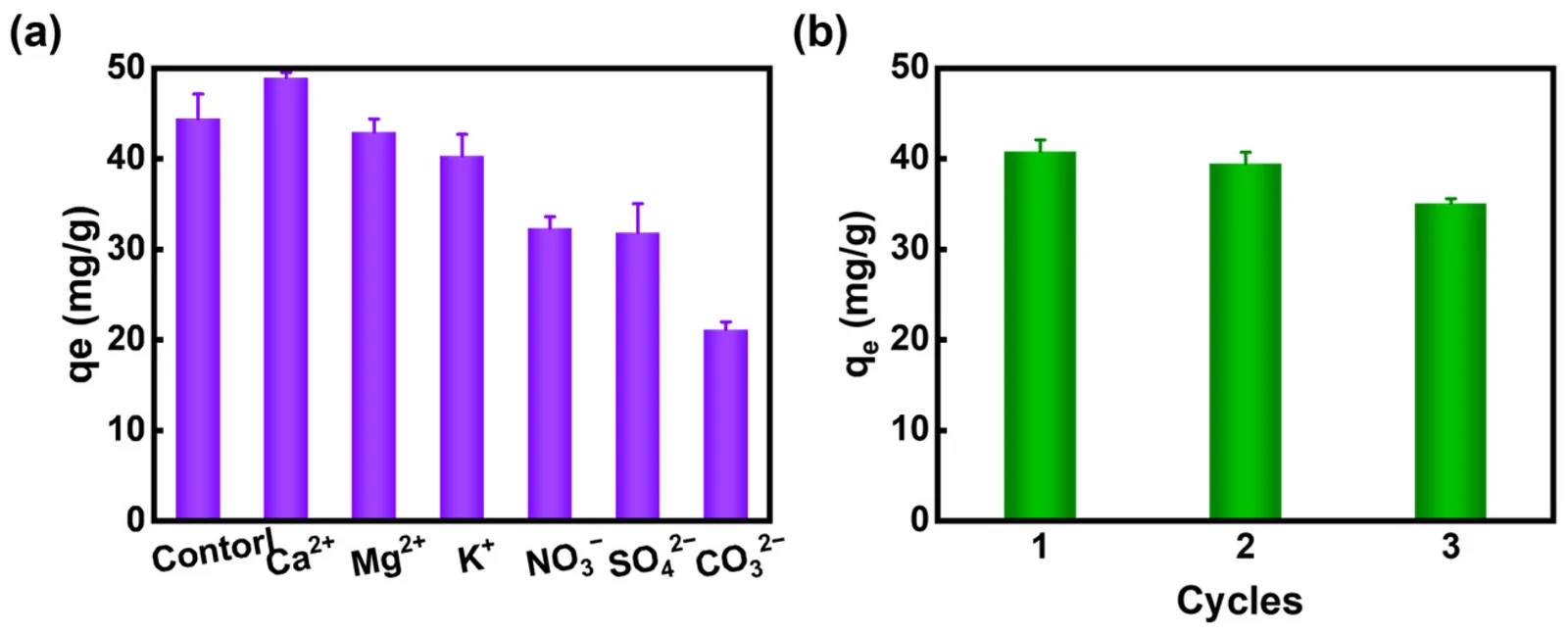
(**a**) Effect of interference substances of NMDG/PEI/UiO-66. (**b**) Reusability of NMDG/PEI/UiO-66.

**Figure 7:**
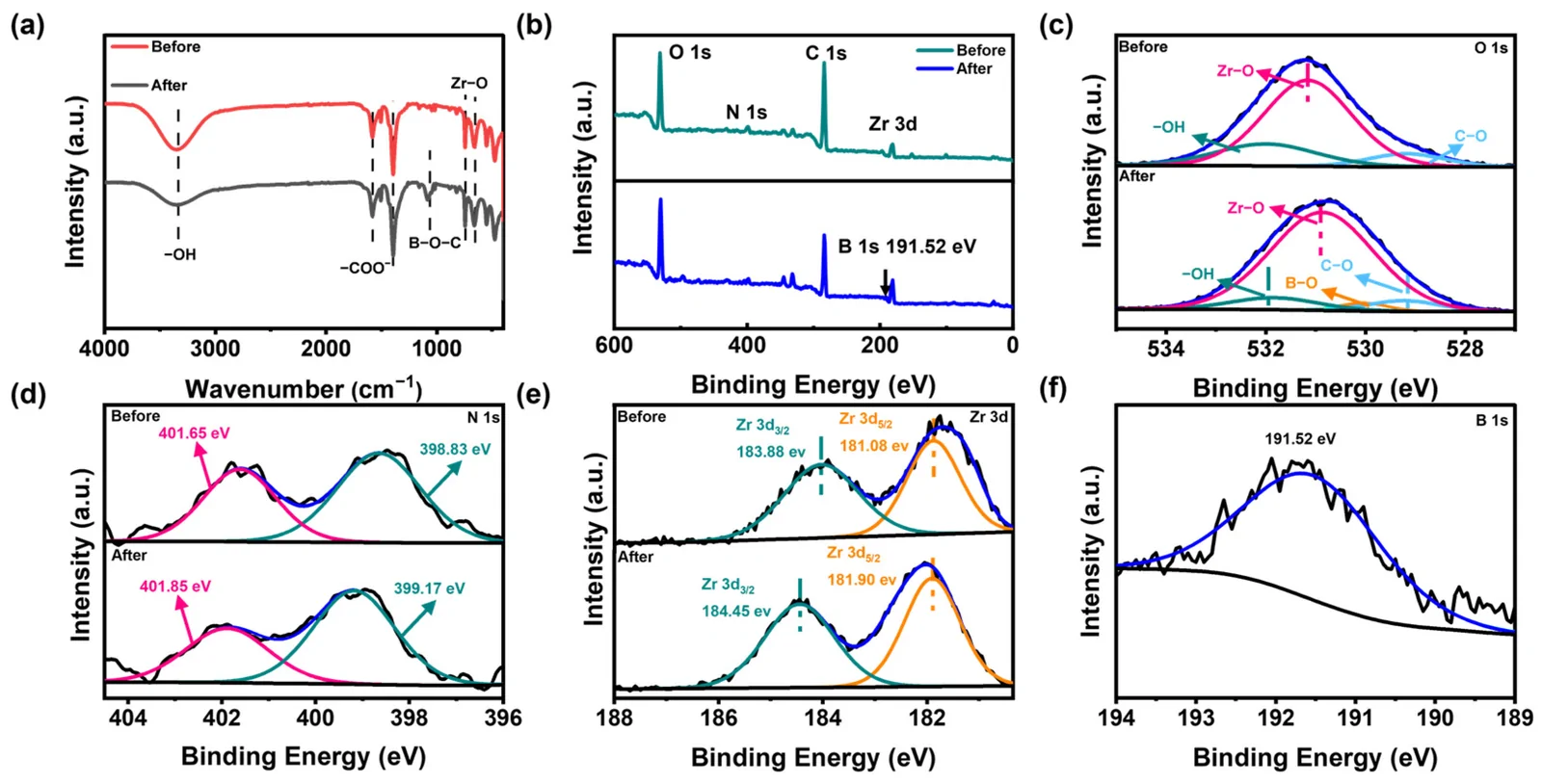
(**a**) FTIR spectra of NMDG/PEI/UiO-66 before and after adsorption. (**b**) XPS survey of NMDG/PEI/UiO-66 before and after adsorption. XPS spectra of (**c**) O 1s, (**d**) N 1s, (**e**) Zr 3d, and (**f**) B 1s.

**Figure 8:**
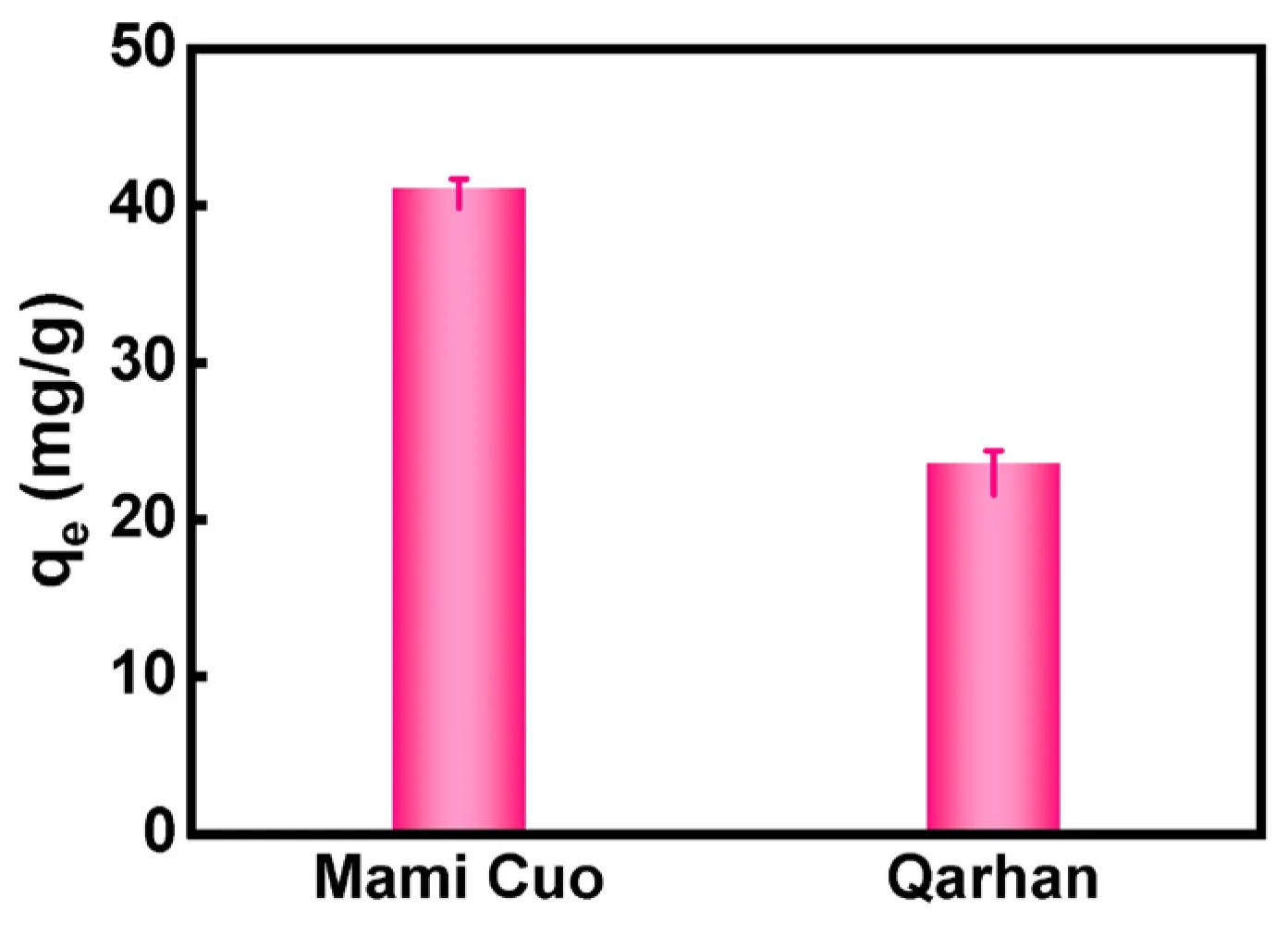
Adsorption of NMDG/PEI/UiO-66 in real brine.

**Table 1:** Comparison of adsorption performance of NMDG/PEI/UiO-66 with other reported boron adsorbents.

	C_0_ (g/L)	pH	Q_m_ (mg/g)	T (°C)	Refs
Fe-LiAl-LDHs	0.5	10	42.07	45	[2]
PP-g-GMA-NMDG	0.4	6	41.65	25	[38]
CNTs@PDA-PEI-NMDG	0.4	9	38.92	25	[19]
MIL-100 (Fe^3+^, Co^2+^)	5	-	43.50	25	[39]
UiO-66	0.5	9	37.26	25	[26]
NMDG/PEI/UiO-66	1.5	9	76.11	25	This work

## Data Availability

The data presented in this study are available upon request from the corresponding author.

## Supplementary Materials

The following supporting information can be downloaded at https://www.mdpi.com/article/10.3390/molecules31193410/s1, Section S1: Apparatus used for characterization of materials; Section S2: Apparatus used for characterization of materials; Figure S1: (a) TG and (b) DTG curves of NMDG/PEI/UiO-66 obtained under a nitrogen atmosphere at a heating rate of 10 °C/min; Table S1: Kinetic fitting parameters; Table S2: Isotherm adsorption fitting parameters; Table S3: Thermodynamic parameters for NMDG/PEI/UiO-66; Table S4: Physicochemical properties of the real brines from Qarhan Salt Lake and Mami Cuo Salt Lake used in this study; Table S5: XPS fitting parameters for NMDG/PEI/UiO-66 before and after boron adsorption; Table S6: Original Ce-qe adsorption data of boron on NMDG/PEI/UiO-66 at 298, 308, and 318 K.
