# Supplementary material for: NMDG-Functionalized UiO-66 via PEI Bridging for Efficient Boron Adsorption in Salt Lake Brine
Source: Molecules. 2026 Sep 24;31(19):3410. doi: 10.3390/molecules31193410 (PMC13665752; doi:10.3390/molecules31193410)
Supplement: Supplementary file 1 [file molecules-31-03410-s001.zip › molecules-4557864-supplementary.pdf]

molecules

MDPI

Supplementary Information

NMDG-Functionalized UiO-66 via PEI Bridging for Efficient Boron Adsorption in Salt Lake Brine

Min Guo [1,2,†], Xunduo Wang [3,†], Leiyang Zhao [3], Yuping Xiao [1,*], Jiaojiao Zhang [1], Chengbing Ma [1,2], Qin Shuai [3] and Shengrui Xu [3,*]

[1] Qaidam Comprehensive Geological and Mineral Exploration Institute of Qinghai Province, Golmud 816099, China; guo.1027@163.com (M.G.); zhang062703@163.com (J.Z.); 18919595628@163.com (C.M.)
[2] Qinghai Provincial Key Laboratory of Salt Lake Resources Exploration and Research in Qaidam Basin, Golmud 816099, China
[3] Faculty of Materials Science and Chemistry, China University of Geosciences, Wuhan 430074, China; 1202520287@cug.edu.cn (X.W.); 1202611396@cug.edu.cn (L.Z.); shuaiqin@cug.edu.cn (Q.S.)
* Correspondence: 18109798908@163.com (Y.X.); xushengrui@cug.edu.cn (S.X.)
† These authors contributed equally to this work.

Section S1. Apparatus used for characterizations of materials

The microstructures of the NMDG/PEI/UiO-66 were characterized by a scanning electron microscope (SEM, SU8010, Hitachi, Tokyo, Japan). X-ray diffraction spectra (XRD, D8 ADVANCE, Bruker AXS, Karlsruhe, Germany) was used to characterize the compositions of NMDG/PEI/UiO-66. Fourier-transform infrared (FTIR) spectra were recorded by a Thermo Nicolet iS50 spectrometer (Thermo Fisher Scientific, Waltham, MA, USA) in the range of 4000-400 cm^{-1}. The surface chemical groups were detected by X-ray photoelectron spectroscopy (XPS, ESCALAB QXi X spectrometer, Thermo Fisher Scientific, Waltham, MA, USA). The BET surface area and pore size distribution were obtained by a nitrogen adsorption/desorption apparatus (Micromeritics ASAP 2460, Atlanta, GA, USA). Thermogravimetric analysis (TGA) was performed using a TG 209 F3 thermogravimetric analyzer (NETZSCH-Gerätebau GmbH, Selb, Germany).

Section S2. Adsorption experiments

The effect of solution pH on adsorption capacity was investigated with 10 mg of adsorbent, 20 mL of 200 mg/L H_3BO_3 solution as the adsorption liquid, and an adsorption time of 12 h. The pH was adjusted using 1 mol/L HCl or NaOH solution. The adsorption was carried out in a constant temperature water bath shaker at 25°C with a rotation speed of 200 r/min.

Dynamic experiment: The dosage of the adsorbent was 10 mg, the adsorption solution was 20 ml of 200 mg/L H_3BO_3 solution, and the adsorption time was 8 hours. Samples were taken at regular intervals to determine the time when saturation adsorption was reached. A constant temperature water bath shaker was used, with an adsorption temperature of 25℃ and a rotation speed of 200 r/min.

The adsorption data were subjected to pseudo-first order kinetic model fitting (Eq. S1) and pseudo-second order kinetic model fitting (Eq. S2) as follows.

Pseudo-first-order: $\mathrm{In}q_e - q_t = \mathrm{In}q_e - k_1 t$ （S1）

Pseudo-second-order: $\frac{t}{q_t} = \frac{1}{k_2 q_e^2} + \frac{t}{q_e}$ （S2）

 https://doi.org/10.3390/molecules31193410

where q_e (mg/g) is the equilibrium adsorption capacity, q_t (mg/g) is the adsorption capacity at time t (min), k_1 (min^{-1}) is a pseudo-first order fitting constant, and k_2 (g mg^{-1} min^{-1}) is a pseudo-second order fitting constant.

In the adsorption isothermal experiment, the amount of adsorbent was 10 mg, the adsorption solution was 20 ml of H_3BO_3 solution with a concentration ranging from 50 to 2000 mg/L, and the adsorption time was 8 hours. Using a constant temperature water bath shaker, the adsorption temperatures were 25, 35, and 45°C, and the rotation speed was 200 r/min.

Thermodynamic experiment: The dosage of the adsorbent was 10 mg, the adsorption solution was 20 ml of 200 mg/L H_3BO_3 solution, and the adsorption time was 8 hours. Using a constant temperature water bath shaker, the adsorption temperature was adjusted to 25, 35, and 45°C, with a rotation speed of 200 r/min. The influence of temperature on the adsorption capacity was studied and the thermodynamic parameters were calculated.

The above data were simulated by different isothermal model, Langmuir (Eq. S4) and Freundlich (Eq. S5):

$$\text{Langmuir model } \frac{C_e}{q_e} = \frac{1}{K_L q_m} + \frac{C_e}{q_m} \quad \text{(S4)}$$

where K_L (L /mg) is the adsorption equilibrium constant of the model and q_m (mg/g) is the maximum adsorption capacity. The model parameters of K_L and q_m were calculated by plotting the C_e/q_e-C_e relationship.

$$\text{Freundlich model:} \log q_e = \frac{1}{n} \log_{C_e} + \log_{K_F} \quad \text{(S5)}$$

where K_F (L mg^{-1}) is the adsorption equilibrium constant for the model. The model parameters of K_F and n were obtained computationally by plotting the log q_e-log C_e relationship.

The corresponding parameters such as enthalpy change (ΔH^{θ}), entropy change (ΔS^{θ}), and Gibbs free energy change (ΔG^{θ}) during the reaction were calculated based on the Vant' Hoff equation using Eq. (S6) and (S7) obtained from the graphs of 1/T and equilibrium constant $\ln K^0$.

$$\text{In}K_C = \frac{\Delta S_{ads}}{R} - \frac{\Delta H_{ads}}{RT} \quad \text{(S6)}$$

$$\Delta G_{ads} = -RT\text{In}K_D \quad \text{(S7)}$$

The effect of interference substance experiment was conducted to study the effects of K^+, Mg^{2+}, Ca^{2+}, SO_4^{2-}, CO_3^{2-}, and NO_3^- on boron adsorption. Sodium salts and chlorides were mixed with the boron acid aqueous solution in their respective forms, with C(B) = 200 mg/L, $C_{(K^+, Mg^{2+}, Ca^{2+}, SO_4^{2-}, CO_3^{2-}, NO_3^-)}$ = 200 mg/L. The adsorbent dosage was 10 mg, the adsorption solution volume was 20 ml, and the adsorption time was 8 hours.

Cyclic experiments were conducted. Each time, the solid-liquid ratio was maintained at 0.5 g/L, and the adsorption duration was 8 hours. 0.5 M NaOH was used for desorption. During the desorption process, the solid-liquid ratio was 1 g/L and the desorption time was 2 hours.

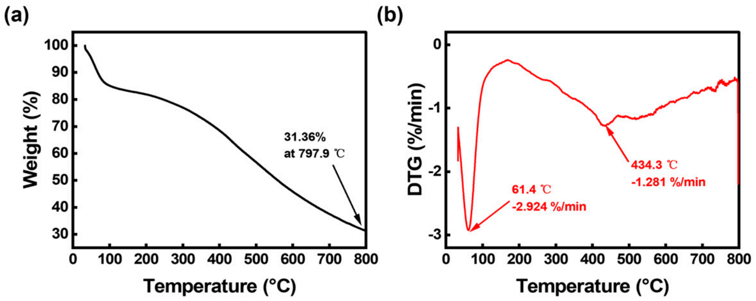

Figure S1. (a) TG and (b) DTG curves of NMDG/PEI/UiO-66 obtained under a nitrogen atmosphere at a heating rate of 10 °C/min.

Table S1. Kinetic fitting parameters.

C_0(mg/L)	Pseudo-first-order			Pseudo-second-order		
	K_1(h^{-1})	Q_e(mg g^{-1})	R^2	K_2(g $mg^{-1}.h^{-1}$)	Q_e(mg g^{-1})	R^2
200	0.384	9.77	0.600	0.0870	40.85	0.997

Table S2. Isotherm adsorption fitting parameters.

T (K)	Langmuir isotherm model			Freundlich isotherm model		
	K_L	Q_m (mg g-1)	R^2	K_F	1/n	R^2
298	0.003	97.59	0.978	4.67	0.413	0.786
308	0.005	69.80	0.988	5.84	0.320	0.790
318	0.007	62.41	0.994	6.33	0.205	0.814

Table S3. Thermodynamic parameters for NMDG/PEI/UiO-66.

T (K)	ΔG^θ (kJ mol^{-1})	ΔH^θ (kJ mol^{-1})	ΔS^θ (J mol^{-1} K^{-1})
298	4.52	-11.44	-53.56
308	5.06		
318	5.09		

Table S4. Physicochemical properties of the real brines from Qarhan Salt Lake and Mami Cuo Salt Lake used in this study.

Parameter	Unit	Qarhan	Mami Cuo
pH	-	8.63	8.95
density	g/cm^3	1.296	1.062
TDS	g/L	420.09	91.27
Cl^-	g/L	306.54	44.76
SO_4^{2-}	g/L	0.187	11.86
CO_3^{2-}	mg/L	-	71.20
HCO_3-	mg/L	-	145.55
Na^+	g/L	3.37	25.73
K_+	g/L	2.96	4.21
Ca^{2+}	g/L	5.37	0.209
Mg^{2+}	g/L	101.46	2.64
B	mg/L	169.36	360.45

Table S5. XPS fitting parameters for NMDG/PEI/UiO-66 before and after boron adsorption.

Region	Component	Assignment	Before BE (eV)	Before FWHM (eV)	After BE (eV)	After FWHM (eV)
O 1s	Zr-O	Zr-O-Zr /Zr-O-C	531.16	2.08	530.87	2.45
	C-O	C-O-H/ C-O-C	529.17	1.81	529.22	1.69
	-OH	Surface hydroxyl	532.00	2.40	531.83	2.07
	B-O	B-O-C	-	-	530.04	1.15
N 1s	-NH-/-NH_2	Free amine	398.83	2.12	399.17	2.06
	$–NH_2^+$	Protonated amine	401.65	1.83	401.85	2.09
Zr 3d	Zr 3d5/2	Zr-O cluster	181.08	1.66	181.90	1.60
	Zr 3d3/2	Zr-O cluster	183.88	1.66	184.45	1.60
B 1s	B-O		-	-	191.52	1.97

Table S6. Original C_e-q_e adsorption data of boron on NMDG/PEI/UiO-66 at 298, 308, and 318 K.

	q_e（mg/g）								
	50 mg/L	100 mg/L	200 mg/L	300 mg/L	400 mg/L	500 mg/L	1000 mg/L	1500 mg/L	2000 mg/L
298 K	15.125	25.755	40.79	53.585	64.295	68.245	72.525	76.11	74.575
308 K	9.373	20.14	32.495	45.6	54.59	56.071	58.27	59.036	61.426
318 K	7.69	16.725	25.265	40.38	48.1	50.96	54.77	53.735	52.68
